# Exploring the interplay between metabolic power and equivalent distance in training games and official matches in soccer: a machine learning approach

**DOI:** 10.3389/fphys.2023.1230912

**Published:** 2023-10-24

**Authors:** Vincenzo Manzi, Cristian Savoia, Elvira Padua, Saeid Edriss, Ferdinando Iellamo, Giuseppe Caminiti, Giuseppe Annino

**Affiliations:** ^1^ Department of Humanities Science, Pegaso Open University, Naples, Italy; ^2^ The Research Institute for Sport and Exercise Sciences, The Tom Reilly Building, Liverpool John Moores University, Liverpool, England, United Kingdom; ^3^ Federazione Italiana Giuoco Calcio (F.I.G.C.), Rome, Italy; ^4^ Department of Human Sciences and Promotion of the Quality of Life, San Raffaele Roma Open University, Rome, Italy; ^5^ Sport Engineering Lab, Department Industrial Engineering, University of Rome “Tor Vergata”, Rome, Italy; ^6^ Department of Rehabilitation Cardiology, IRCCS San Raffaele Pisana, Rome, Italy; ^7^ Department of Clinical Science and Translational Medicine, University of Rome Tor Vergata, Rome, Italy; ^8^ Centre of Space Bio-Medicine, Department of Systems Medicine, Faculty of Medicine and Surgery, University of Rome “Tor Vergata”, Rome, Italy

**Keywords:** soccer (football), training load (TL), metabolic power (MP), equivalent distance (ED), machine learning (ML)

## Abstract

**Introduction:** This study aimed to explore the interplay between metabolic power (MP) and equivalent distance (ED) and their respective roles in training games (TGs) and official soccer matches. Furthermore, the secondary objective was to investigate the connection between external training load (ETL), determined by the interplay of metabolic power and equivalent distance, and internal training load (ITL) assessed through HR-based methods, serving as a measure of criterion validity.

**Methods:** Twenty-one elite professional male soccer players participated in the study. Players were monitored during 11 months of full training and overall official matches. The study used a dataset of 4269 training games and 380 official matches split into training and test sets. In terms of machine learning methods, the study applied several techniques, including K-Nearest Neighbors, Decision Tree, Random Forest, and Support-Vector Machine classifiers. The dataset was divided into two subsets: a training set used for model training and a test set used for evaluation.

**Results:** Based on metabolic power and equivalent distance, the study successfully employed four machine learning methods to accurately distinguish between the two types of soccer activities: TGs and official matches. The area under the curve (AUC) values ranged from 0.90 to 0.96, demonstrating high discriminatory power, with accuracy levels ranging from 0.89 to 0.98. Furthermore, the significant correlations observed between Edwards’ training load (TL) and TL calculated from metabolic power metrics confirm the validity of these variables in assessing external training load in soccer. The correlation coefficients (r values) ranged from 0.59 to 0.87, all reaching statistical significance at *p* < 0.001.

**Discussion:** These results underscore the critical importance of investigating the interaction between metabolic power and equivalent distance in soccer. While the overall intensity may appear similar between TGs and official matches, it is evident that underlying factors contributing to this intensity differ significantly. This highlights the necessity for more comprehensive analyses of the specific elements influencing physical effort during these activities. By addressing this fundamental aspect, this study contributes valuable insights to the field of sports science, aiding in the development of tailored training programs and strategies that can optimize player performance and reduce the risk of injuries in elite soccer.

## 1 Introduction

Soccer is a physically demanding sport that requires athletes to possess excellent fitness and a range of skills, including endurance, explosive power, and strength, to meet the high-energy demands of the game (Stølen et al., 2005). Elite soccer players experience elevated heart rate and oxygen uptake demands during official matches because they participate in high-intensity activities such as sprinting, jumping, and changing direction at high speeds (Stølen et al., 2005; Bangsbo et al., 2006). Additionally, soccer involves intricate, unpredictable movement patterns during matches, with about 70% of the time consisting of low-intensity activities like jogging or walking (Bangsbo et al., 2006; Rampinini et al., 2007a; Di Salvo et al., 2010). In soccer, these low-intensity activities serve as essential recovery periods, allowing players to recuperate after engaging in high-intensity actions. This dynamic pattern mirrors the stop-and-start nature of soccer matches, where players must efficiently manage their energy expenditure to sustain performance throughout the game (Bangsbo, 1994). In recent years, training games (TGs) have become an essential training tool in soccer due to their ability to simulate real match scenarios and improve physical qualities specific to soccer matches. Training games (TGs) in soccer refer to a broad category of practice exercises and drills strategically designed to replicate real match scenarios and situations. These drills encompass various technical and tactical aspects of the game, including passing, dribbling, and positioning. A significant advantage of TGs is that players have more chances to touch the ball and make decisions, which can enhance their technical abilities and decision-making skills (Clemente et al., 2021; Savoia et al., 2021). While several studies have shown positive evidence for the benefits of TGs in soccer training, performance during such training exercises may not be a reliable indicator of physical performance during official matches. Studies have shown that TGs can provide intense training, as demonstrated by high values of heart rate, blood lactate levels, and the number of accelerations per minute (Sarmento et al., 2018). Still, they may only partially replicate the tactical and positional demands of soccer matches, as cautioned by Dellal et al. (2012), who found improvements in physical performance but not full replication of official match demands. For instance, a study by Dalen et al. (2021) revealed that 4 vs. 4 and 6 vs. 6 Small-Sided Games (SSGs) did not meet the high-intensity running and sprinting performance levels observed in official matches. Additionally, as the pitch size per player decreased (10 vs. 10 > 7 vs. 7 > 5 vs. 5), there were reductions in total distance covered, distances run at high speeds (>14.4 km·h-1), absolute maximum velocity, and the number of accelerations and decelerations. However, there was an increase in the number of moderate accelerations and decelerations, as well as the total number of velocity changes, with smaller pitch dimensions. Moreover, it has been documented that the number of accelerations during SSG training surpasses that observed during actual match play (Castellano et al., 2013). On the other hand, a 6-a-side SSG can replicate the high-intensity demands of an 11-a-side full-sized pitch, but only if the area per player in the SSGs is roughly half the size of a full-sized pitch. In alignment with this rationale, Riboli et al. (2020) demonstrated that adjusting SSGs by varying the area per player, introducing a goalkeeper, or implementing specific rules can heighten or diminish the position-specific demands concerning the desired external load outcomes. Furthermore, it has been established that the game format can alter the physiological and activity demands encountered during SSGs. Employing an identical pitch size (40 × 20 m) but altering the number of players (from 3 to 5-aside) can influence physical responses to the exercise, including factors like heart rate, blood lactate levels, and perceived exertion (RPE), among others. Therefore, a comprehensive understanding of the physiological demands of soccer matches and TGs is critical in evaluating the physical performance of professional players. Recent technological advancements in sports science, like local position measurement (LPM), semi-automated computerized tracking systems, and global positioning systems (GPS) accurately assess players’ performance during matches and training sessions by measuring various physiological external load parameters. These parameters include energy expenditure, metabolic power (MP), and acceleration/deceleration patterns (Osgnach et al., 2010; Osgnach and di Prampero, 2018). It is worth noting that several studies have raised concerns about the applicability of the Osgnach formula in team sports, particularly in capturing the full scope of metabolic demands inherent in such dynamic and complex activities (Buchheit et al., 2015). The formula’s limitations in adequately representing the metabolic demands of team sports have been a subject of debate within the sports science community. However, it is essential to emphasize that the choice of MP calculation method is often context-dependent, contingent upon research objectives and available resources. The Osgnach formula, despite its limitations, has been widely used in prior sports science research, including soccer, and has offered valuable insights into metabolic demands (Brochhagen and Hoppe, 2022). Moreover, the application of the Osgnach formula could be complemented by additional metrics and machine learning techniques. This multi-faceted approach would allow for a more comprehensive analysis considering metabolic and kinematic parameters. Comparing performance parameters recorded during match-play with those obtained during training and exercise helps sports scientists provide performance references for training optimization (di Prampero and Osgnach, 2018; Hill-Haas et al., 2011). One way to assess physical performance in soccer is through MP, which indirectly measures the rate at which energy is produced and consumed during physical activity based on the player’s acceleration and velocity (Hoppe et al., 2017; Osgnach and di Prampero, 2018). It is pertinent to note that the calculation of MP using GPS monitoring technology differs from the estimation of MP through the measurement of oxygen uptake, as highlighted in several studies (Buchheit et al., 2015; Savoia et al., 2020). These studies have raised questions about the comparability and accuracy of MP values obtained through GPS monitoring and metabolic chart-based estimations, particularly in team sports settings like soccer. To estimate accurate MP, calculating oxygen consumption via a metabolic chart can be considered a precise method (Savoia et al., 2020). Similarly, GPS monitoring devices can track various factors related to a player’s physical performance, including energy expenditure, metabolic power, and biomechanical movements such as acceleration and deceleration (Rechenchosky et al., 2017). Additionally, GPS technology can measure equivalent distance (ED) as another metric in soccer, which quantifies the distance a player covers while adjusting for changes in speed and direction. It could provide a more accurate measure of the physical demands of a match or training session than simply measuring the total distance covered (Pillitteri et al., 2023a). ED considers that different movements have varying metabolic demands and thus contribute differently to a player’s overall physical workload (Osgnach et al., 2010; Caldeira et al., 2022). It was demonstrated that the equivalent distance index (EDI), the ratio between the ED and the total distance covered in a match or TGs, showed a strong correlation with accelerations and decelerations events; for this reason, both metabolic and traditional approaches based on time spent in arbitrarily chosen running speed categories should be used together for load monitoring in professional soccer players. Specifically, the same research has indicated metabolic power events and the EDI as variables capable of differentiating the player’s characteristics more clearly, considering their playing position (Guerrero-Calderón et al., 2022). Despite the common use of physiological and kinematic parameters, such as heart rate, blood lactate, accelerations, decelerations, and speed, to measure soccer-related movements during official matches and TGs, to our knowledge, no study has explored the MP and ED of both game types simultaneously using these two parameters concomitantly. Integrating MP and ED parameters can provide a more comprehensive understanding of the exercise intensity during TGs and official matches. Integrating both MP and ED parameters in external load monitoring is crucial because it offers a more comprehensive understanding of exercise intensity during Training Games (TGs) and official soccer matches. While MP and ED represent different aspects of physical performance, they both respond to accelerations, which significantly determine energy expenditure and overall intensity. For instance, when players undergo rapid accelerations, MP and ED values increase substantially, reflecting the higher energy demands associated with such movements. Conversely, during periods of reduced acceleration, MP and ED values decrease, indicating lower energy expenditure. By considering both MP and ED alongside acceleration data, practitioners can gain a more nuanced view of player performance. This integrated approach allows coaches and sports scientists to identify the overall intensity of the activity and the specific moments within a game or training session where high-intensity accelerations occur. These insights are valuable for tailoring training programs, optimizing player performance, and minimizing the risk of overuse injuries. Thus, this study aims to elucidate the dynamic relationship between MP and ED, providing practical examples of how these parameters interact to enhance external load monitoring in elite soccer. Given the complexity of the data in analyzing the physiological and kinematic parameters of soccer matches and TGs, machine learning (ML) algorithms can be highly beneficial in this study for several reasons. They can efficiently handle large and diverse data sets, identify patterns and relationships that traditional statistical methods may miss, and continuously adapt and learn from new data to improve performance prediction and decision-making. Over the past decade, ML has emerged as a popular approach in sports science research for analyzing complex data sets in sports performance analysis. ML is a powerful tool that can help sports scientists and coaches gain valuable insights into player performance and make data-driven decisions to enhance athletic performance (Rico-González et al., 2023). Therefore, the main goal of this study is: 1) to examine the interplay between MP and ED and their relative contribution to TGs and official soccer matches; 2) the secondary aim of this study was to examine the relationship between the external training load (ETL), determined by the interplay between MP and ED, and the internal training load (ITL) estimated using the HR-based method, which was used as a criterion validity measure. It is hypothesized that TGs and official matches could show different patterns of MP and ED. Integrating physiological and kinematic parameters could provide a better understanding of the nature of the exercise intensity during these two types of activities.

## 2 Materials and methods

### 2.1 Experimental approach to the problem

This study is a retrospective analysis of existing data, which aims to distinguish between TGs and official matches in soccer based on MP and EDI. Specific precisely of elite-standard soccer players participating in the Italian Premier Division championship (series A) was followed during the 2021–2022 preseason (i.e., over an entire season 11 months). The study utilized ML techniques to analyze data collected previously on the physiological and kinematic demands of soccer activities. The data were divided into two groups: TGs and official matches. Four different ML methods were applied to the data to determine whether they could accurately discriminate between the two groups based on MP and EDI. The metabolic power metrics were developed with the aim of precisely describing the physical demands placed on players during a soccer match or a training session (di Prampero et al., 2015). The combination of metabolic power and kinematic parameters provides an integrated measure of acceleration and velocity, making it a more accurate indicator of the physiological effort soccer players exert during training and match play (Osgnach et al., 2010). To calculate the MP, researchers utilized the following formulas (Minetti et al., 2002; di Prampero et al., 2005):

$$MP=EC\times \vec{v}$$

Where EC represents the energetic cost of running uphill at the inclination obtained from the running acceleration vector, and $\vec{v}$ is the instantaneous velocity. In addition, to better calculate the metabolic intensity of the game, some authors suggest incorporating both the ED and the EDI (Guerrero-Calderón et al., 2021; Guerrero-Calderón et al., 2022). The ED is the distance an athlete would have run at a constant pace on grass using the total energy spent over the match, while the EDI represents the ratio between the ED and the total distance covered during the game. It is important to note that the EDI is an indirect estimate of the metabolic intensity of the game, and a high index suggests a significant contribution of anaerobic stores due to high acceleration bouts. To calculate the ED and EDI, researchers utilized the following formulas (Osgnach et al., 2010):

$$ED=\frac{W}{\left({EC}_{c}\times KT\right)}$$

$$EDI=\frac{\mathrm{ED}}{\mathrm{TD}}$$

Where W is the total energy expenditure measured in joules per kilogram, EC_c_ is the energetic cost of running at a constant pace on flat compact terrain assumed to be 3.6 J kg^-1^·m^-1^, and KT is the grassy terrain constant. ED is the equivalent distance measured in meters, and TD is the total distance covered in meters. Based on the reported data, the average ratio between ED and the distance covered during soccer matches was approximately 1.20 (Osgnach et al., 2010). However, it should be noted that this ratio varied widely among players, with individual ratios ranging from about 1.15 to 1.35 (di Prampero et al., 2015). Various factors influenced these ratios, including the player’s role on the field. To our knowledge, previous studies have not yet utilized the product of the metabolic power and equivalent distance index to calculate the external training load intensity. Therefore, our study calculated the ETL by multiplying the MP by the EDI. The product of the MP and the EDI can be referred to as the intensity of the external load I). Thus, the ETL is given by the product of volume, terms of training time V) and power I), or:

$$ETL=\int V\left(t\right)dt\times \int I\left(t\right)dt$$

$$I\;\left(\mathrm{AU}\right)=MP\times EDI$$

Where:

V = training time (in minutes).

MP = metabolic power (W·kg^-1^).

EDI = equivalent distance index.

To investigate the respective impact of MP and EDI on determining the ETL, we took the natural logarithm of each variable separately. We calculated the ratio between the logarithm of MP and EDI and the logarithm of intensity I). Specifically, we used the following formulas:

$$Ratio=\frac{\ln\left(MP\right)}{\ln\left(I\right)}$$

$$Ratio=\frac{\ln\left(EDI\right)}{\ln\left(I\right)}$$

These calculations allowed us to gain insights into the impact of the MP and EDI on ETL and better understand the relationship between these variables and physical effort. The calculation of the ratio between the natural logarithms of MP and EDI, each divided by the natural logarithm of intensity I), allows us to understand the impact of MP and EDI on External Training Load (ETL) and the relationship between these variables. This ratio-based approach helps us dissect the individual contributions of Metabolic Power (MP) and Equivalent Distance Index (EDI) to the overall ETL. By comparing the logarithmic ratios of MP/I and EDI/I, we can assess how changes in MP and EDI affect the intensity of the external load I). A higher ratio for MP/I suggests that variations in MP have a more pronounced effect on the ETL, indicating that metabolic demands significantly influence training intensity. Conversely, a higher ratio for EDI/I indicates that changes in EDI have a more significant impact on ETL. In addition, to examine the relationship between internal and ETL, we utilized Edwards’ objective HR-based method (Edwards, 1993) as the criterion validity and compared the ETL to it. This method involves multiplying the accumulated duration (in minutes) spent in five HR zones by a coefficient specific to each zone (50%–60% of HR_max_ = 1, 60%–70% of HR_max_ = 2, 70%–80% of HR_max_ = 3, 80%–90% of HR_max_ = 4, 90%–100% of HR_max_ = 5) and then summing the results. Edwards’ TL has been validated and used as a criterion measure of ITL in various studies examining different methods of monitoring training load (Foster et al., 2017; Izzo and Giovannelli, 2017; Çalı et al., 2020). This allowed us to evaluate the association between the two measures and gain insight into the validity of the ETL as an indicator of internal load. In this study, the Edwards’ TL was calculated from heart rate (HR) data collected during each training session over an entire season.

### 2.2 Participants

This study involved 21 male professional soccer players in the Italian Serie A league. The characteristics of the players are presented in Table 1. All the players were active members of one team in the Serie A championship. The players were categorized based on their playing position, with central backs (CB, n = 4), side backs (SB, n = 4), midfielders (MF, n = 6), wingers (WI, n = 3) and forwards (FW, n = 4). All the players had at least 5 years of competitive experience in the premiership. They were monitored over an entire season (11 months), including pre-season, in-season, and all official matches (Serie A matches and Italian Cup matches), which took place from July until May during the 2021–2022 seasons. The squads systematically played in a 4-3-1-2 formation model, with four defenders (two FBs and two CBs), 3 MFs, one WI, and three FWs. All players underwent the same training modality sessions seven times a week throughout the pre-season, with a friendly match played on Thursday or during the weekend. Training sessions were mainly focused on technical-tactical skill development, and fitness training sessions were performed with a single training modality (i.e., no other exercises) during the pre-season. During the championship, players trained six times weekly, with a match played on the weekend. Friendly and cup matches usually occurred on Thursday and Wednesday, respectively. Each professional player gave informed consent about the research purposes of using the results observed during their usual training sessions and official matches. All participants were informed about the study protocol and gave their informed consent to participate. This study was approved by the Internal Research Board of the University of Rome “Tor Vergata.” All the procedures involved in this study were in accordance with the Declaration of Helsinki.

**TABLE 1:** Anthropometric and physiological data according to playing position.

Variables	Central back (n = 4)	Side back (n = 4)	Midfielder (n = 6)	Winger (n = 3)	Forward (n = 4)
Age (years)	27 ± 5	26 ± 2	26 ± 4	27 ± 2	28 ± 6
Height (cm)	189 ± 2	182 ± 2	177 ± 5	180 ± 4	183 ± 5
Body weight (kg)	83.7 ± 4.8	80.1 ± 3.0	76.4 ± 3.6	77.5 ± 3.7	78.4 ± 2.1
Body fat (%)	10.8 ± 2.0	11.2 ± 2.8	11.8 ± 3.1	11.0 ± 2.4	11.4 ± 1.9
Max heart rate (bpm)	187 ± 7	189 ± 6	184 ± 10	180 ± 5	186 ± 5
$\dot{V}O2_{\max}$ (ml·kg^-1^·min^-1^)	56.7 ± 2.2	59.8 ± 5.3	59.4 ± 3.0	58.3 ± 5.0	54.5 ± 4.9

### 2.3 Anthropometric and physiological parameters

Each participant’s height and body weight were measured using a stadiometer (Seca 213, Hamburg, Germany) and a digital scale (Tanita BC-418, Tokyo, Japan), respectively. Body fat percentage was estimated using a bioelectrical impedance analyzer (Tanita BC-418, Tokyo, Japan). The $\dot{V}O2_{\max}$ was assessed using a progressive maximal test completed on a 400-m athletic track, until exhaustion (Léger and Boucher, 1980; Berthoin et al., 1999). For every 20 m, a cone was positioned as a reference. After an acoustic signal, the subjects performed the incremental field test, starting from 8.0 km h^-1^, with the speed then increased by 0.5 km h^-1^ every minute. The end of the trial was considered when the player twice failed to reach the next cone at the required time (objective evaluation) or he felt unable to cover another interval at the dictated speed (subjective evaluation). During the test, players were verbally encouraged by the test leaders and coaches to provide maximal effort in the late stages of the test. Achievement of $\dot{V}O2_{\max}$ was considered as the attainment of at least 2 of the following criteria: a) a plateau in $\dot{V}O2$ despite increasing speeds, b) a respiratory exchange ratio above 1.10, c) an HR ± 10 b min^-1^ of age-predicted HR_max_ (208–0.7 age) (di Prampero, 1986; Howley et al., 1995; Tanaka et al., 2001). Expired gases were analyzed using a breath-by-breath automated gas analysis system (K4b^2^; COSMED, Rome, Italy) (Eisenmann et al., 2003; Duffield et al., 2004). The highest HR measured in the maximal incremental test was used as HR_max_. Criteria for HR_max_ achievement were attainment of subjective and visual exhaustion, blood lactate concentrations higher than 8 mmol L^-1^, and HR plateau achievement despite speed increments (Åstrand, 1952; Inbar et al., 1994).

### 2.4 Video match analysis

Match analysis was conducted using the STATS SportVU^®^ system, a validated multicamera video analysis tool from STATS LLC in Chicago, United States. The STATS SportVU^®^ system is a sophisticated and widely used technology in sports analytics, especially soccer. It utilizes multiple high-definition cameras installed at various angles around the stadium to track players and the ball at rates up to 25 Hz, with a measurement accuracy determined by the Technical University of Munich (TUM) to have a typical error of 2.7% for total distance (Linke et al., 2018). K-Sport provided raw data in Cartesian coordinates, and the primary data were smoothed at 5 Hz (Gaudino et al., 2013). STATS SportVU uses HD cameras, advanced software and statistical algorithms to extract and process player (X, Y) and ball (X, Y, Z) coordinates (Linke et al., 2020). Roof-level cameras captured player movements during matches, and the data were analyzed using STATS Viewer and STATS Dynamix, both provided by STATS Perform, to create a dataset of each player’s physical and technical performance. For subsequent analysis, players who played the entire match or were substituted no more than 5 min before the final whistle (≥85 min of play) were included. Specific software (https://www.gpexe.com) was used to download and analyze data extracted from STATS SportVU, including metabolic power (W kg^-1^), equivalent distance index (%), and other metabolic-energetic metrics. Data were filtered according to the theoretical model based on an energetic approach where the energy cost of accelerations and decelerations plays a central role, as di Prampero et al. (2005) suggested.

### 2.5 GPS technology

The metabolic power parameters during each training sessions were evaluated using high-frequency devices: GPS pro^2^ at 18-Hz (GPEXE^©^, Exelio srl, Udine, Italy). The reliability, accuracy, and validity of the GPEXE^©^ system have been reported elsewhere (Nagahara et al., 2017; Hoppe et al., 2018). This level of detail allows for a granular assessment of players’ running patterns, changes of direction, and speed profiles during training sessions and matches. This technology provides trainers with real-time data on players’ training performance, allowing for objective decisions based on actual data. The system can calculate over 300 metrics in real-time and transfers data accurately and promptly during training sessions. Moreover, it enables three-dimensional tracking of individual players or teams over time, assessing different positional workloads, determining training intensities, and monitoring changes in players’ physiological demands. All GPS devices were activated 15 min before data collection to ensure high-quality signals and enable satellite lock. Signal quality was assessed based on the number of connected satellites (11 ± 1.4) and the horizontal dilution of precision (0.9 ± 0.1) according to Witte and Wilson. (2004).

### 2.6 Training games (TGs)

Integrating TGs into soccer training provides a consistent and flexible approach for enhancing players’ technical, tactical, and physical skills. As a popular alternative to traditional drills, TGs offer engaging game-specific scenarios that can benefit elite soccer players looking to improve their performance (Beato et al., 2023a; Zlojutro et al., 2023). However, it can be challenging to assess the physiological and performance demands of TGs and ensure that they closely match the needs of official matches. To address this issue, we compared the physiological and performance data collected during the TGs to that of official matches. This allowed us to determine whether the TGs provided a reliable simulation of match play and whether they could effectively contribute to the development of players’ functional capacities in soccer players. In this study, we investigated the interaction between MP and ED and their respective impact in TGs (small and medium games, average pitch ratio per player 125 m^2^, range 54–187 m^2^, see Table 2) and official soccer matches by analyzing the external and ITL in both. Similar work was carried out by Beato et al. (2023b), using the rating of perceived exertion (RPE) as players’ internal load. The TGs formats based on various factors, including the number of players, game duration, number of sets, length of the break between sets, and dimensions of the playing field, are presented in Table 2. During the TGs, the players were instructed to play high intensity and score as many goals as possible. The coach provided minimal feedback, letting the players take ownership of the game and encouraging a competitive atmosphere. We ensured that all players wore the same GPS unit during each training session to minimize measurement inaccuracies.

**TABLE 2:** Table comparing the performance of different machine learning models (KNN, Random Forest, SVM, Decision tree) in terms of AUC, classification accuracy (CA), F1 score, precision, and recall for different soccer player positions (CB, SB, MF, WI, and FW).

Model (KNN)	AUC	CA	F1	Precision	Recall
Central back	0.96	0.98	0.98	0.98	0.98
Side back	0.95	0.97	0.97	0.97	0.97
Midfielder	0.95	0.95	0.95	0.95	0.95
Winger	0.94	0.95	0.95	0.94	0.95
Forward	0.93	0.94	0.94	0.94	0.94
**Model (Random Forest)**	**AUC**	**CA**	**F1**	**Precision**	**Recall**
Central back	0.98	0.98	0.98	0.98	0.98
Side back	0.97	0.98	0.98	0.98	0.98
Midfielder	0.96	0.96	0.96	0.96	0.96
Winger	0.94	0.95	0.95	0.95	0.95
Forward	0.96	0.96	0.96	0.96	0.96
**Model (SVM)**	**AUC**	**CA**	**F1**	**Precision**	**Recall**
Central back	0.94	0.98	0.98	0.98	0.98
Side back	0.91	0.97	0.97	0.97	0.97
Midfielder	0.89	0.95	0.95	0.95	0.95
Winger	0.89	0.94	0.94	0.94	0.94
Forward	0.89	0.94	0.94	0.94	0.94
**Model (Decision tree)**	**AUC**	**CA**	**F1**	**Precision**	**Recall**
Central back	0.96	0.97	0.97	0.97	0.97
Side back	0.96	0.97	0.97	0.97	0.97
Midfielder	0.95	0.94	0.94	0.94	0.94
Winger	0.94	0.94	0.94	0.94	0.94
Forward	0.95	0.93	0.93	0.94	0.93

AUC, Area under the ROC curve; CA, Classification accuracy; F1, F1 score, balancing precision and recall; Precision, Precision, the fraction of correct positive predictions; Recall, Recall, the fraction of true positives correctly classified.

### 2.7 Data analysis

In our study, we utilized several supervised ML methods for dataset classification, including the K-Nearest Neighbors (KNN), Decision Tree (DT), Random Forest (RF), and Support-Vector Machine classifiers (SVM) (Rao, 1948; Cover and Hart, 1967; Rossi et al., 2022). These methods are known for their ability to generate graphical outputs that are easy to interpret and provide visualizations of the impact of multi-dimensional features on class variables. The K-Nearest Neighbors classifier (KNN) is a non-parametric and lazy learning algorithm that identifies the k nearest neighbors to a given data point and classifies it based on the most common class among its neighbors (Rao, 1948). The Decision Tree classifier (DT) is a tree-based classification algorithm that builds a decision tree by recursively partitioning the feature space into subsets based on the value of the most informative feature (Quinlan, 1986). The Random Forest classifier (RF) is an ensemble learning algorithm that combines multiple decision trees to improve the accuracy and stability of the classification model (Breiman, 2001). The Support-Vector Machine (SVM) is a discriminative classifier that works by finding the hyperplane that maximizes the margin between the classes in the feature space (Cortes and Vapnik, 1995). These ML methods were selected for their ability to provide interpretable graphical outputs that visualize the impact of multi-dimensional features on class variables. We validated our classifiers with a 3-fold stratified cross-validation strategy (Friedman, 2001; Hastie et al., 2001). The association between training load, as determined by integrating MP and ED, and HR-based TL (Edwards’ TL) was analyzed using the Pearson correlation coefficient (Cohen, 1988). The strength of correlation was qualitatively classified according to Hopkins as follows: trivial r < 0.1, small 0.1 < r < 0.3, moderate 0.3 < r < 0.5, large 0.5 < r < 0.7, very large 0.7 < r < 0.9, nearly perfect r ≈ 0.9, and perfect r = 1 (Hopkins, 2002; Batterham and Hopkins, 2006). Automated preprocessing and advanced analysis, including the K-Nearest Neighbors (KNN), Decision Tree (DT), Random Forest (RF), and Support-Vector Machine (SVM) classifiers, were performed using Orange 3 (Demšar and Zupan, 2013). Unless otherwise stated, the results are presented as the mean ± SDs throughout the text, and 95% confidence intervals (CIs) are presented where appropriate. Before any parametric statistic was calculated, the assumption of normality was tested with a Shapiro-Wilk test in each variable (Shapiro and Wilk, 1965). The significance level was set at *p* < 0.05.

## 3 Results

### 3.1 The interplay between MP and ED on TGs and official matches in soccer

**FIGURE 1:**
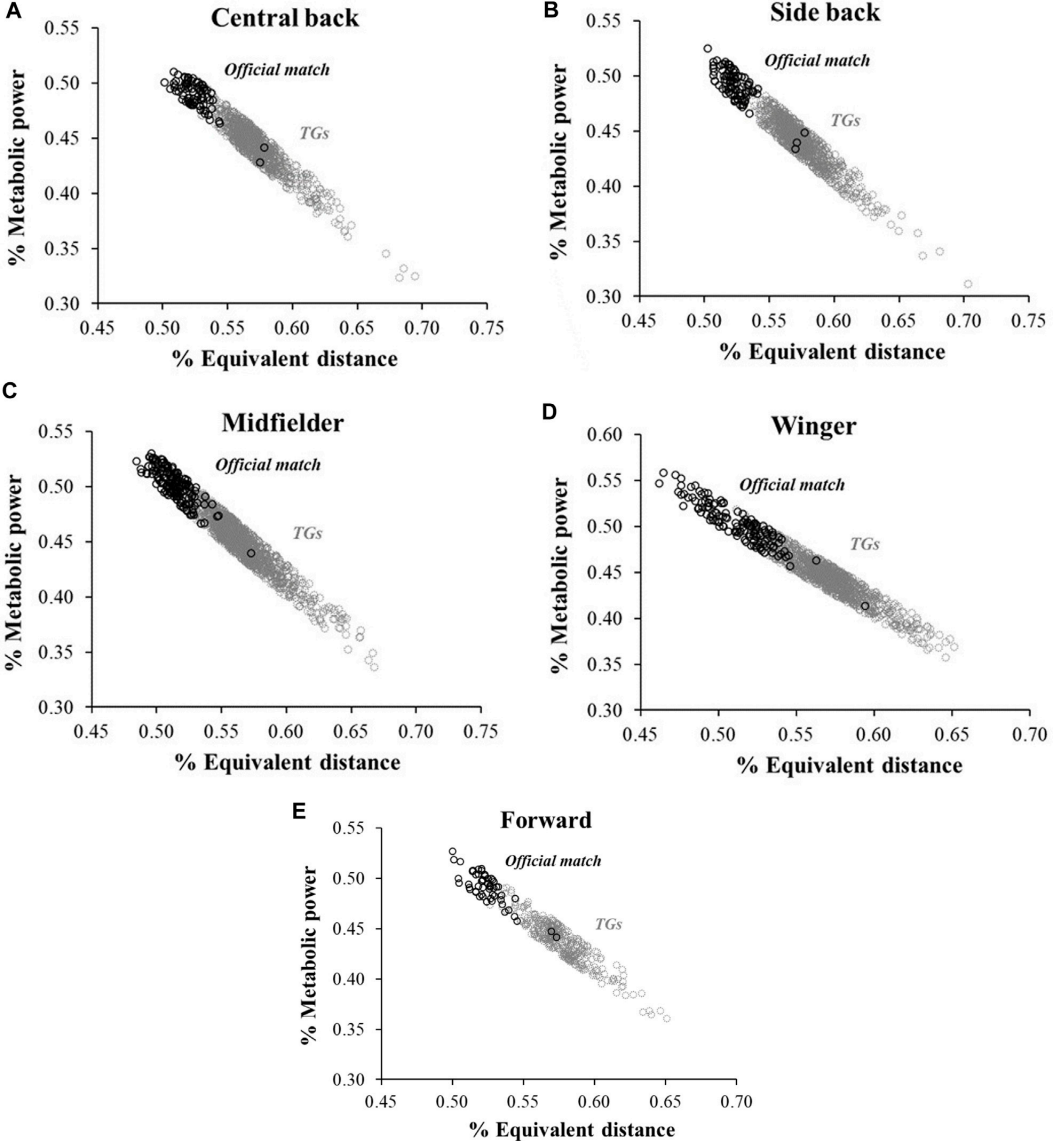
The scatter plot provides a 2-dimensional visualization for the accuracy of the KNN model in order to illustrate the differences between TGs and official soccer matches. Labels **(A–E)** represents all soccer player positions.

To assess the ability of MP and ED to distinguish between the two types of soccer activities, we used a dataset of 4.269 TGs and 380 official matches. Table 4 shows the results of our study, which used four different ML methods (KNN, DT, RF, and SVM) to classify TGs and official matches based on MP and ED. All the methods achieved high levels of accuracy, with AUC values ranging from 0.90 to 0.96. The accuracy (range from 0.89 to 0.98) (accuracy measures the proportion of correctly classified instances over the total number of cases in the dataset. It is a standard metric used for binary and multi-class classification tasks and provides an overall indication of how well the model predicts the correct class); F1 score (the harmonic mean of precision and recall is used to balance the trade-off between precision and recall. It provides a single score that represents the model’s performance on both precision and recall) ranging from 0.93 to 0.98; precision, (measures the proportion of instances classified as positive, e.g., training games or official matches that were correctly classified. It is useful when the cost of false positives is high) range from 0.94 to 0.98, and recall, (known as sensitivity or true positive rate, measures the proportion of positive instances, in our case, correctly classified samples of training games or official matches that the model correctly identified. It is useful when the class distribution is imbalanced, as it focuses on correctly identifying the positive instances) ranging from 0.93 to 0.98, further demonstrating the high level of accuracy in recognizing the differences between the two soccer activities based on MP and ED. In addition to Table 4, we included Figure 1 to visually represent the accuracy, F1 score, precision, and recall of the KNN model. These graphs provide further evidence for the high level of accuracy in distinguishing between TGs and official matches based on an integration between MP and ED. In our results the variability in machine learning model performance across different player roles is a notable observation. Specifically, defensive positions such as central and side back consistently exhibited robust model performance, with consistently high scores across various metrics. In contrast, the midfielder, winger, and forward roles displayed slightly lower but still discernible performance levels (Figure 1; Table 4). The average intensity, calculated as an interplay between MP and ED, of the TGs was observed to be 1.20 ± 0.29 AU, with the highest intensity level being 2.72 AU. During official matches, the average intensity was 1.19 ± 0.21 AU, with the highest intensity level being 3.05 AU. Notably, there were no significant differences in intensity between TGs and official matches (mean difference 0.01 AU; 95% CI -0.02–0.37).

### 3.2 The relationship between the external training load (ETL) and the internal training load (ITL)

The correlation between Edwards’ TL and the TL based on metabolic power metrics was examined, and the results are presented in Table 3. The analysis revealed large to very large correlations, with correlation coefficients ranging from 0.59 to 0.87 (*p* < 0.001). Finally, our study did not find a statistically significant correlation between the MP and EDI in TGs and official soccer matches (r = 0.017; 95% CI -0.051 to 0.085, *p* > 0.05).

**TABLE 3:** Individual correlations between Edwards’ TL and the TL based on metabolic power metrics; all individual correlations were statistically significant (*p* < 0.01).

Subjects	Total training (n)	r	95% CI
S1	129	0.70	0.60 to 0.78
S2	199	0.59	0.49 to 0.68
S3	246	0.74	0.67 to 0.79
S4	128	0.82	0.75 to 0.87
S5	179	0.74	0.67 to 0.80
S6	143	0.85	0.80 to 0.89
S7	218	0.78	0.72 to 0.83
S8	254	0.69	0.62 to 0.75
S9	207	0.83	0.78 to 0.87
S10	128	0.85	0.79 to 0.89
S11	126	0.80	0.72 to 0.85
S12	155	0.83	0.77 to 0.87
S13	174	0.85	0.80 to 0.88
S14	212	0.87	0.84 to 0.90
S15	169	0.80	0.73 to 0.85
S16	207	0.82	0.76 to 0.86
S17	186	0.82	0.76 to 0.86
S18	199	0.75	0.69 to 0.81
S19	114	0.61	0.49 to 0.72
S20	113	0.76	0.67 to 0.83
S21	206	0.74	0.67 to 0.80
Max	254	0.87	0.84 to 0.90
Min	113	0.59	0.49 to 0.68
**Team**	**3692**	**0.75**	**0.73 to 0.76**

### 3.3 The relationships between area per player (ApP) and TGs intensity

No significant correlation was found between the pitch size per player and TGs intensity (r = 0.06, 95% CI -0.12 to 0.23; *p* > 0.05).

## 4 Discussion

### 4.1 The interplay between MP and ED on TGs and official matches in soccer

Utilizing machine learning techniques to analyze extensive datasets of physiological and kinematic variables within the soccer domain, this study aimed to uncover distinct patterns specific to soccer activities and disparities that might be elusive when examining individual studies or limited sample sizes. The results showed that the four different ML methods successfully discriminated between TGs and official matches based on MP and EDI (Table 4; Figure 1), achieving high levels of accuracy with AUC values ranging from 0.90 to 0.96. The accuracy, F1 score, precision, and recall further demonstrated the ability to distinguish between the two types of soccer activities. The study also found no significant correlation between pitch ratio per player and TGs intensity, with an average intensity of 1.20 ± 0.29 AU observed in TGs and no significant differences in intensity between TGs and official matches. These findings are consistent with previous literature on the physiological demands of TGs and official matches in soccer (Casamichana et al., 2012; Beato et al., 2023a; Castillo-Rodríguez et al., 2023).

**TABLE 4:** Training games formats based on dimensions of the playing field and number of players.

Training game	Number of players (n)	Lenght (m)	Width (m)	Pitch size (m^2^)	Pitch ratio per player (m^2^)	Set (n)	Duration (min)	Rest (s)
Training game 4vs.4	8	30	32	960	120	3	3	90
Training game 5vs.5	10	40	42	1680	168	4	4	60
Training game 5vs.5	10	36	30	1080	108	4	4	60
Training game 5vs.5	10	30	36	1080	108	4	3	60
Training game 5vs.5	10	32	40	1280	128	4	4	60
Training game 5vs.5	10	40	32	1280	128	4	4	60
Training game 6vs.6	12	40	38	1520	127	4	4	60
Training game 6vs.6	12	40	44	1760	147	4	4	60
Training game 6vs.6	12	40	42	1680	140	2	4	60
Training game 6vs.6	12	32	40	1280	107	4	4	60
Training game 6vs.6	12	38	40	1520	127	4	4	60
Training game 6vs.6	12	34	40	1360	113	3	4	60
Training game 7vs.7	14	40	42	1680	120	4	4	60
Training game 7vs.7	14	40	36	1440	103	4	3	60
Training game 7vs.7	14	40	50	2000	143	4	4	60
Training game 8vs.8	16	36	50	1800	113	4	4	60
Training game 8vs.8	16	40	46	1840	115	4	4	60
Training game 8vs.8	16	36	40	1440	90	5	3	45
Training game 8vs.8	16	40	36	1440	90	5	3	45
Training game 8vs.8	16	40	52	2080	130	2	4	60
Training game 9vs.9	18	44	64	2816	156	4	4	60
Training game 9vs.9	18	40	50	2000	111	2	6	60
Training game 9vs.9	18	40	44	1760	98	5	3	30
Training game 9vs.9	18	44	73	3212	178	4	4	60
Training game 9vs.9	18	44	50	2200	122	4	4	60
Training game 9vs.9	18	50	52	2600	144	2	5	60
Training game 9vs.9	18	40	52	2080	116	4	4	60
Training game 10vs.10	20	50	52	2600	130	2	4	60
Training game 10vs.10	20	44	50	2200	110	2	4	60
Training game 10vs.10	20	52	50	2600	130	4	4	60
Training game 10vs.10	20	52	72	3744	187	4	4	60
Training game 10vs.10	20	50	72	3600	180	2	8	60
Training game 10vs.10	20	50	44	2200	110	3	4	60
Training game 10vs.10	20	50	68	3400	170	5	4	60
Training game 10vs.10	20	50	60	3000	150	4	4	60
Training game 10vs.10	20	40	32	1280	128	4	4	60
Training game 10vs.10	20	34	52	1768	88	2	8	60
Training game 10vs.10	20	30	36	1080	54	1	8	60
Training game 10vs.10	20	50	73	3650	183	5	4	60
Training game 10vs.10	20	34	48	1632	82	2	6	60
Training game 10vs.10	20	36	48	1728	86	4	6	60

### 4.2 The relationships between area per player (ApP) and TGs intensity

Several studies have reported that TGs elicit higher metabolic demands than official matches, with higher mean heart rates, oxygen uptake, and blood lactate concentrations (Buchheit et al., 2010; Dellal et al., 2012; Rebelo et al., 2012). In contrast, official matches have been found to result in greater total distance covered and higher intensity of activity at high speeds (distances covered in running speed >19.6 km h^-1^) compared to TGs (Mohr et al., 2003; Bradley et al., 2013; Rebelo et al., 2013; Beato et al., 2023b; Gualtieri et al., 2023). It is extremely important to evaluate the exposure to the training load by considering ApP as a meaningful task constraint to increase the internal and ETL experienced by soccer players, independent of format and age group (Clemente et al., 2023). Of course, the manipulation of the area per player (ApP), the presence of the goalkeeper, or the design-specific rules contribute to increase or decrease the position-specific demands concerning the desired external load outcomes (Castellano and Casamichana, 2013; Lacome et al., 2018; Riboli et al., 2020). However, this study’s novel approach of using ML analysis to distinguish between TGs and official matches based on MP and EDI provides new insights into the differences between these two types of soccer activities (TGs and official matches, Jaspers et al., 2018; Rico-González et al., 2023). Furthermore, the significant correlations between Edwards’ TL and the TL based on MP metrics support the validity of using both parameters to measure ETL in soccer (Table 3). This finding is consistent with previous research demonstrating the usefulness of metabolic power metrics for monitoring external training load in soccer (Osgnach et al., 2010; Manzi et al., 2014).

### 4.3 The relationship between the external training load (ETL), and the internal training load (ITL)

Our analysis revealed large to very large correlations between Edwards’ TL and the TL based on MP metrics. In contrast, no significant correlation was found between MP and EDI in both TGs and official soccer matches. Overall, the correlation between external and internal load is a crucial concept for sports science and exercise physiology, and the use of MP and EDI as indicators of external load in soccer can provide valuable insights for coaches and trainers in monitoring and managing training loads (Rossi et al., 2019; Pillitteri et al., 2023b; Impellizzeri et al., 2023). It is worth noting that previous studies (Di Mascio et al., 2015; Gaudino et al., 2015; Akenhead and Nassis, 2016) used slightly different external load metrics to correlate with Edwards’ internal TL, including total distance, high-intensity distance, sprinting frequency, high-speed distance, and the number of accelerations and decelerations derived from GPS data. However, all the above studies found significant correlations between internal and external training load measures, indicating that ETL metrics can be useful for monitoring and managing training loads in soccer. Moreover, the present study adds to the existing literature by investigating the relationship between MP and EDI as external load metrics and Edwards’ internal TL. Understanding the relationship between external and internal loads, as measured by MP and ED, is essential for coaches and trainers to effectively monitor and manage the physical demands of TGs and official matches to optimize performance and try to decrease injury risk. This research furthermore allows us to expand what has already been studied previously by Rossi et al. (2018) and Vallance et al. (2020) on injuries forecasting through ML. For instance, if the internal load (i.e., the physiological stress placed on the athlete’s body) is disproportionately higher than the external load (i.e., the physical demands of the activity), it may suggest that the athlete is unable to handle the demands of the sport or training program (Manzi et al., 2022). This situation could increase the risk of overuse injuries, fatigue, and other negative outcomes. On the other hand, if the external load is too high relative to the internal load, it may indicate that the athlete is not being challenged enough, and the training program may need to be adjusted to elicit a greater physiological response. An important finding is that no significant correlation was found between MP and EDI, suggesting that these parameters capture different aspects of soccer activity. This result is in line with previous studies that have highlighted the importance of considering multiple parameters to capture the physiological demands of soccer fully (Bradley et al., 2013; Carling et al., 2015; Bradley and Ade, 2018; Impellizzeri et al., 2023). To put these results into context, previous studies have found that TGs are generally less intense (e.g.,. distance per minute) and demanding than official matches, with lower values for variables such as total distance covered per minute, high-intensity running, and sprinting (Dellal et al., 2011; Lacome et al., 2018; Savoia et al., 2021). However, it is worth noting that these studies used different methodologies and variables to measure intensity and demands, which makes it difficult to compare their findings directly with those of the present study. Moreover, some studies have reported conflicting results regarding the differences between TGs and official matches (Mohr et al., 2003; Rampinini et al., 2007b). Against this backdrop, the results of this study provide valuable insights into the potential use of MP and ED as reliable indicators of the differences between TGs and official matches. The ApP and intensity are two factors to consider when designing the TGs, as they can affect the players’ physiological and performance demands, technical and tactical abilities, and overall experience during the training process (Kelly and Drust, 2009; Casamichana et al., 2018; Riboli et al., 2020; Castillo-Rodríguez et al., 2023; Clemente et al., 2023). Interestingly, no significant correlation was observed between pitch ratio per player and TGs intensity. One plausible explanation for this lack of association is that smaller playing areas impose a greater EDI because increases the acceleration contribution, whereas a large pitch ratio per player tends to have the opposite effect (i.e., decrease). Conversely, if players have more ApP, they tend to run at higher speeds when they do get the opportunity. This increase in speed is directly linked to an increase in MP (Reference). Even though there were no significant differences in intensity (the product of the MP and the EDI can be referred to as the intensity of the external load) between TGs and the official matches, it is possible that the contribution of MP and ED to the intensity differs between these two types of activities. Classification models were used in this study to better account for the specific factors contributing to intensity in each type of activity. These models demonstrated that, despite similarities in overall intensity, the contribution of MP and ED to the intensity might differ between TGs and official matches (Table 4; Figure 1). Therefore, it is important to carefully consider these factors when comparing the physiological demands of different types of activities. In other words, although the overall intensity may be similar, the underlying factors contributing to that intensity may differ between TGs and the official matches. This is a crucial factor to consider when comparing the physiological demands of different activities and highlights the need for more detailed analyses of the specific factors contributing to intensity. For example, it is possible that the different formats of TGs used in the study may have emphasized certain aspects of the game, such as technical skills or tactical positioning, which may not fully reflect the demands of a real match. Similarly, differences in the duration and intensity of TGs compared to official matches could affect the specific physiological adaptations that occur in response to each type of activity. Therefore, it is important to consider the specific demands of each activity and ensure that training programs are designed to target those demands in a way that closely replicates the physiological and metabolic demands of real matches. In this context, our study’s findings have practical implications for coaches and trainers, who can use MP and EDI to design training programs that closely replicate the demands of official matches. This is consistent with prior research (Di Mascio et al., 2015; Malone et al., 2018; Savoia et al., 2021) and underscores the importance of monitoring and managing training loads using these indicators.

Our study suggests that coaches and trainers should aim to replicate not only the overall intensity of official matches but also the specific distribution and percentages of metabolic power (MP) and equivalent distance index (EDI) that occur during those matches. For instance, if our analysis shows that a significant portion of MP in official matches comes from high-speed running, it becomes crucial to ensure that training games (TGs) also generate a similar percentage of MP from these activities. This means designing TGs that mimic the distribution of effort seen in actual matches.

In practical terms, it implies that if 40% of the MP in official matches arises from high-speed actions, then TGs should also aim to replicate this 40% proportion in their training drills. This ensures that players are exposed to the overall intensity and the same relative distribution of physical efforts. This approach goes beyond intensity and focuses on recreating the precise percentages of MP and EDI developed during actual game situations. Another non-negligible point of this research concerns the possibility of interpreting the data of MP and EDI as indicators of a more muscular effort or less oriented towards neuromuscular aspects; all this could implement recent studies with ML on fatigue and recovery to the specificity of the external load, helping to optimize training and customize loads and recovery also in team sports (Mandorino et al., 2022; Pillitteri et al., 2023c; Pillitteri et al., 2023d). By considering physiological and kinematic parameters, our study highlights the need for a holistic approach to evaluating exercise intensity during different activities (Reference). The limitations of the Osgnach formula in fully capturing the extensive metabolic demands associated with the dynamic and multifaceted nature of team sports have been debated within the scientific community. It is essential to recognize that specific research objectives and practical considerations influence the choice of the MP calculation method. Finally, while acknowledging the criticism of the Osgnach formula’s suitability for team sports, this study integrated the formula as part of a broader set of metrics to assess exercise intensity. The inclusion of MP, EDI, and machine learning techniques aimed to provide a more holistic understanding of the differences between training games (TGs) and official matches in soccer.

## 5 Limitations

Some limitations of this study should be considered when interpreting the results. Firstly, the study focused only on TGs and official soccer matches, and the results may need to be generalizable to other types of soccer activities or sports. Secondly, the study used a retrospective data analysis, and there was no control over the training or match conditions, which may have introduced biases and confounding factors that could affect the results. Thirdly, the study did not investigate the relationship between external load and performance outcomes, which is essential for understanding the practical implications of the findings. Finally, the main limitation of this study is the use of convenience sampling, aware that ML models depend upon the amount of dataset (Rico-González et al., 2023); consequently, this investigation should be regarded as a case study.

## 6 Conclusion

Despite these limitations, the study provides valuable insights into the potential use of MP and EDI as reliable indicators of the differences between TGs and official matches in soccer. Our results demonstrate that using MP and EDI can help coaches tailor their training programs to match official matches’ bioenergetic and muscular demands. By filling this gap in knowledge, researchers can shed light on the similarities and differences between these game formats and help coaches and trainers optimize their training strategies to improve player performance and reduce the risk of injury.

Further studies are needed to confirm and extend these findings to other types of soccer activities and sports.

## Data Availability

The original contributions presented in the study are included in the article/supplementary material, further inquiries can be directed to the corresponding author.
